# Hypoxia-responsive circRNAs: A novel but important participant in non-coding RNAs ushered toward tumor hypoxia

**DOI:** 10.1038/s41419-022-05114-y

**Published:** 2022-08-01

**Authors:** Benzheng Jiao, Shanshan Liu, Hongguang Zhao, Yuying Zhuang, Shumei Ma, Chenghe Lin, Jifan Hu, Xiaodong Liu

**Affiliations:** 1 Key Laboratory of Radiobiology (Ministry of Health), School of Public Health, Jilin University, Changchun, China; 2 Department of Nuclear Medicine, The First Hospital of Jilin University, Changchun, China; 3 Department of Hematology, The First Hospital of Jilin University, Changchun, China; 4 Stem Cell and Cancer Center, The First Hospital of Jilin University, Changchun, Jilin China; 5 Platform for Radiation Protection and Emergency Preparedness of Southern Zhejiang, School of Public Health and Management, Wenzhou Medical University, Wenzhou, China; 6 Stanford University Medical School, Palo Alto Veterans Institute for Research, Palo Alto, CA USA

**Keywords:** Cancer microenvironment, Long non-coding RNAs

## Abstract

Given the rapid developments in RNA-seq technologies and bioinformatic analyses, circular RNAs (circRNAs) have gradually become recognized as a novel class of endogenous RNAs, characterized by covalent loop structures lacking free terminals, which perform multiple biological functions in cancer genesis, progression and metastasis. Hypoxia, a common feature of the tumor microenvironments, profoundly affects several fundamental adaptive responses of tumor cells by regulating the coding and non-coding transcriptomes and renders cancer’s phenotypes more aggressive. Recently, hypoxia-responsive circRNAs have been recognized as a novel player in hypoxia-induced non-coding RNA transcriptomics to modulate the hypoxic responses and promote the progression and metastasis of hypoxic tumors. Moreover, via extracellular vesicles-exosomes, these hypoxia-responsive circRNAs could transmit hypoxia responses from cancer cells to the cells of surrounding matrices, even more distant cells of other organs. Here, we have summarized what is known about hypoxia-responsive circRNAs, with a focus on their interaction with hypoxia-inducible factors (HIFs), regulation of hypoxic responses and relevance with malignant carcinoma’s clinical features, which will offer novel insights on the non-coding RNAs’ regulation of cancer cells under hypoxic stress and might aid the identification of new theranostic targets and define new therapeutic strategies for those cancer patients with resistance to radiochemotherapy, because of the ubiquity of tumoral hypoxia.

## Facts

1. Some hypoxia-responsive circRNAs are induced by HIFs (especially HIF-1α) in hypoxia, while others are not.
2. Hypoxia-responsive circRNAs reversibly regulate HIFs to facilitate tumor adaptations to hypoxia stress.
3. Hypoxia-responsive circRNAs play key roles in the several hypoxic responses of cancer to facilitate proliferation and metastasis.
4. Hypoxia-responsive circRNAs have relevance with several clinical features of cancer, which will serve as novel diagnostic and prognostic biomarkers.

## Introduction

The “seed and soil theory” hypothesis considers that the tumor microenvironment (TME) is of fundamental importance in terms of tumor proliferation, metastasis, immune evasion and chemoradiotherapeutic resistance [1]. Oxygen deficiency or hypoxia has become the most common and important microenvironmental characteristic in rapidly growing solid tumors that exhibit high level of tumor proliferative and metabolic rates, and poor vascularization. Thus, the rapidly growing tumor cells invoke essential but complex adaptive mechanisms to cope with hypoxia, involving hundreds or thousands of coding and non-coding genes [2, 3].

**Fig. 1:**
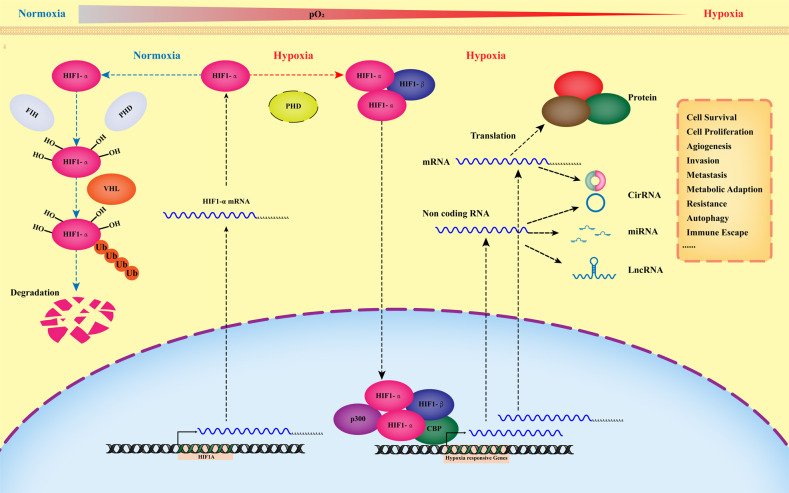
HIF-1α activates downstream coding and non-coding genes transcription in response to hypoxia. Under normoxic conditions, HIF-1α subunits are hydeoxylated by prolyl hydroxylase domain proteins (PHDs) and other prolyl hydroxylases. Hydroxylated HIF-1α subunits are recognized by von Hippel–Lindau (VHL) proteins that target HIF-1α for subsequent ubiquitination and proteasomal degradation. During hypoxia, the hydroxylation reactions are reduced, resulting in HIF-1α accumulation, dimerization with HIF-1β, binding to target genes and activation of such genes via recruitment of p300/CBP and formation of the transcription initiation complex that mediates various hypoxic responses of cancer.

Upon hypoxic conditions, tumor cells initiate the hypoxia-inducible factors (HIFs) complex as the central mediator to modulate fundamental adaptive responses to such microenvironmental stress, although HIFs-independent adaptive responses are also in play. In animal cells, HIFs, as the basic member protein of bHLH/PAS (helix-loop-helix/PER-ARNT-SIM) family, usually form specific heterodimeric complexes between the HIFα and HIFβ subunits. The α subunits of HIFs are mainly subdivided as HIF-1α, HIF-2α and HIF-3α, encoded by HIF1A, HIF2A and HIF3A separately; whereas the β subunits of HIF1 (HIF1B; also known as ARNT) are encoded by ARNT1 and ARNT2. Additionally, HIF-1α and HIF-2α can transcribe independent but overlapping sets of target genes when hypoxia develops [3, 4] (Fig. 1).

Under normoxic conditions, HIF-1α subunits are hydroxylated by a family of dioxygenases (such as PHD1, 2 and 3). The hydroxylated HIF-1α subunits are rapidly identified by the VHL (E3 ubiquitin ligase) and degraded via the ubiquitin-proteasome pathway (Fig. 1a). When hypoxia develops, the HIF-1α subunits accumulate rapidly because that the PHD dioxygenase activity is inhibited, and then they are translocated from cytoplasm to the nucleus, wherein they interact with HIF-1β, CBP (CREB-binding protein) and p300 to form the HIF-1 transcriptional complex, which finally binds to the promoter regions of HIF-1α target genes to trigger a series of cellular hypoxia adaptations, including enhanced cellular proliferation and angiogenesis, decreased apoptosis, increased autophagy, invasion and metastasis, et al. In addition to the protein-coding transcriptome, more and more research has shown that the non-coding transcriptome also responds to hypoxia and plays various critical roles in cancer progression and metastasis under such circumstances [2, 4, 5] (Fig. 1).

The ENCODE (Encyclopedia of DNA Elements) project’s data indicates that less than 2% of human genome encodes protein, while the remaining 98% can be transcribed into various different non-coding RNAs, most of which don’t possess protein-coding potential. Based on their sizes and structures, the non-coding RNAs are generally subdivided into 3 categories: 1) small non-coding RNAs (<200 nucleotides), such as microRNA, transfer-RNAs, small nuclear/nucleolar RNAs and piwi-interacting RNAs; 2) long non-coding RNAs (>200 nucleotides) and 3) circular RNAs. These non-coding RNAs are often deregulated in cancer tissues and play key roles in cancer formation, progression, metastasis, as well as the responses to different survival stresses including hypoxia [2, 3, 6, 7] (Fig. 1).

Here, we will summarize our current knowledge on the regulatory roles of hypoxia-responsive non-coding RNAs, particularly circRNAs, with a focus on their reciprocal regulation with HIFs, regulation of hypoxic responses and relevance with the clinical features of malignant carcinoma, which may make them serve as molecular markers for disease diagnosis, prognosis and evaluation of therapeutic effects.

## Hypoxia-responsive non-coding rnas: a family of powerful players in terms of hypoxia regulation

The most common type of small non-coding RNAs, microRNAs (miRNAs), is a class of single-stranded, endogenous small non-coding RNAs with 20–25 nucleotides in length. In the cytoplasm, these miRNAs and other proteins are recruited by the Argonaute proteins to form the RNA-induced silencing complexes (RISCs) with their target mRNAs. The complexes promote degradation of the target mRNAs or repress their translation via full or partial complementarity. Recently several scholars have profiled the global miRNAs expression patterns in hypoxic tumors [2, 7]. Such hypoxia-responsive miRNAs can be significantly upregulated by hypoxia in a HIF-dependent manner, because hypoxia response elements (HREs) exist in the promoter regions of their gene locus. For example, miR-155, miR-21, miR-424 and miR-210 are significantly upregulated by transcription factors HIF-1α during hypoxia [8–11]. In addition, the expression or stabilization of HIF1α or HIF2α can also be affected by the complex network of miRNAs, which can directly bind to the 3′UTR of HIF1α or HIF2α mRNAs or indirectly affect the actions of their regulatory proteins (VHL or PHDs), such as miR-199a and miR-155 [10, 12]. (For more information and details, please refer to these reviews [2, 7]).

Long non-coding RNAs (lncRNAs), longer than 200 nucleotides, constitute another large and heterogeneous class of non-coding RNAs, and mainly include long intronic ncRNAs, long intergenic RNAs (lincRNAs), antisense RNAs (asRNAs), pseudogenes, transcribed ultra-conserved regions (T-UCRs), and enhancer RNAs (eRNAs) [3, 7, 13]. These lncRNAs play critical roles in expression of genes at multiple regulatory levels, including epigenetic, transcriptional, and post-transcriptional levels [7]. Bioinformatics analyses and next-generation RNA sequencing technologies have revealed many lncRNAs, including hypoxia-responsive lncRNAs (HRLs) that can be divided into two groups. Firstly, there are HRLs that serve as HIFs’ effectors in promoting cell growth or inhibiting cell death. For example, HOTTIP is upregulated by HIF-1α via binding to the HREs in HOTTIP’s promoter region, increasing the epithelial-mesenchymal transition (EMT) and metastasis of glioma by the miR-101/ZEB1 axis [14]. Secondly, there are HRLs that directly or indirectly modulate the HIFs proteins such as aHIF-1α, lncRNA-ROR, and lncRNA-p21 [15, 16] (For more information and details, please refer to these reviews [3, 7, 13]).

## CircRNAs: a novel class of non-coding rna with covalent loop structures lacking free terminals

CircRNAs constitute one of the newest classes of non-coding RNAs, whose discovery was greatly aided by novel RNA-seq technologies and bioinformatic analyses. Increasing evidences indicate that circRNAs are present in many eukaryotic cells and possess numerous important properties and functions, which make circRNAs as the hotspot of non-coding RNA research field recently. Here, we will review the recent works on hypoxia-responsive circRNAs, including their reciprocal interactions with HIFs, their roles played in tumor hypoxic adaptation and their relevance with clinical features of malignant tumors.

**Fig. 2:**
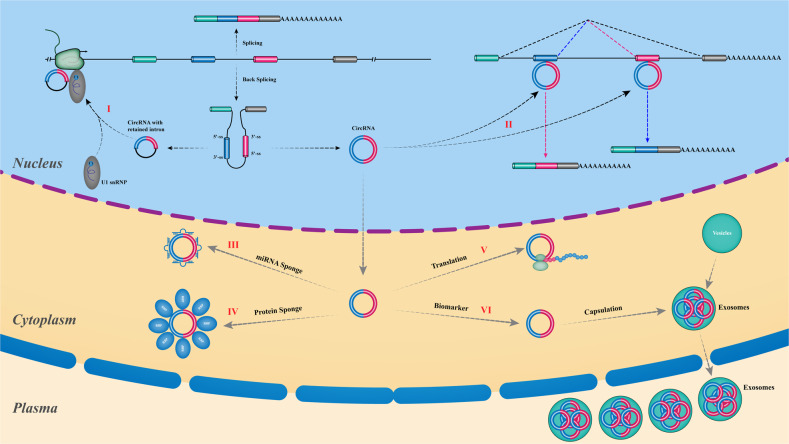
Biogenesis and potential mechanisms of endogenous circRNAs in gene expression regulation. I: Regulation of parental genes transcription via combination with U1 RNP complexs; II: Modulating splicing of linear mRNA counterparts. III: Decoying miRNA as sponges. IV: Serving as scaffolds on which proteins assemble. V: Translating into peptides. VI: Secreted in exosomes that may serve as useful biomarkers.

Circular RNAs were initially found as a viroid in RNA (ribonucleic acid) viruses or endogenous RNA splicing products in eukaryotes cells in the beginning [17, 18]. Subsequently, various forms of circular RNAs have been found to be generated by distinct mechanisms, for example, circular formats of small nucleolar RNAs (snoRNAs), circular RNA intermediates created during rRNA processing, and permuted tRNAs with rearranged segments, et al. [19]. Until 2012, the ubiquity and abundance of circRNAs in eukaryotes were identified at various developmental stages and under various physiological conditions [20]. Based on their genomic origins, circRNAs can be mainly divided into four categories: 1) intergenic circRNAs: derived from intervals between two genes; 2) exonic circRNAs (ecircRNAs): generated predominantly from back-spliced exons; 3) intronic circRNAs (ciRNA): derived from introns; 4) exonintron circRNAs (EIciRNA): circularized between exons and introns [20, 21]. Most known circRNAs are produced via back-splicing of the exons of precursor mRNAs (pre-mRNAs) with a downstream 5′ splice site (ss) joined and ligated with an upstream 3′ ss by a 3′-5′ phosphodiester bond at the junction site. Below, circRNAs refers to exonic circRNAs in the following (Fig. 2).

Given their special circular covalently bonded structure, circRNAs possess some special characteristics. They mainly include: (1) Stability: circRNAs are more stable than their linear analogs, because they are more resistant to RNase R; (2) Abundance: a single host gene can generate into multiple circRNA isoforms via alternative splicing; (3) Conservation: circRNAs exist in diverse species from bacteria to human being; (4) Specificity: the expressive spectrum of circRNAs differ among cell types, tissues, and developmental stages of the same organism [1, 20].

Similar to lncRNAs, circRNAs also play critical roles in multiple regulatory levels, which can be summarized as follows: (1) CircRNAs serves as specific miRNA “sponges” or “reservoirs”: some can absorb miRNAs to prevent such them from engaging in 3′-UTR complementary pairing regions of target mRNA, whereas others can either conversely stabilize or disassemble into miRNAs; (2) CircRNAs interact with proteins as “scaffolds”: some derived from RNA-binding protein (RBP) genomic site exibit the conserved binding sites of their parental host protein, while others serve as dynamic scaffolds facilitating the assembly of diverse proteins; (3) CircRNAs play as regulators of gene transcription and expression: circEIF3J and circPAIP2 can assemble with U1 snRNP in the nucleus to enhance the Pol II RNA polymerase-mediated transcription activity of their parental genes; (4) CircRNAs serve as protein/peptide translators: some feature internal ribosomal entry site (IRES) elements or prokaryotic ribosome binding sites in their structures and can thus be translated into proteins or peptides in a cap-independent manner [1, 19–21] (Fig. 2).

## Hypoxia-responsive circrnas: novel participants in hypoxia-induced non-coding RNA transcriptomics

The roles played by circRNAs in hypoxic cancer cells remain poorly known, although hypoxia-induced other non-coding RNA transcriptomics (miRNAs and lncRNAs) have been extensively studied. However, since hypoxia-responsive circRNAs were identified in endothelial cells in 2015, many such circRNAs have been transcribed in hypoxic tumor cells [22]. With RNA-sequencing, bioinformatic analyses and subsequent quantitative validation (e.g. qRT-PCR), several hypoxia-responsive circRNAs have been identified in hypoxia-exposed gastric cancer (MKN-28 cells): 24 circRNAs have been found to be upregulated and 21 downregulated, compared to the normoxia control [23]. In another study, high throughput RNA-seq identified 558 circRNAs in hypoxic lung adenocarcinoma cells (A549 cells), of which 35 circRNAs were differentially upregulated and 30 downregulated, and circRNA-miRNA networks were computationally constructed [24]. Using a consolidated computational pipeline (find_circ and CIRCexplorer), Di Liddo A et al. identified ∼12000 circRNAs were expressed during hypoxia of three different cancer cell lines: A549, HeLa and MCF-7. Meanwhile, hypoxia significantly changed the levels of 64 circRNAs in a cell type-dependent manner [25].

### HIFs-dependent regulation of hypoxia-responsive circRNAs in tumors

**Fig. 3:**
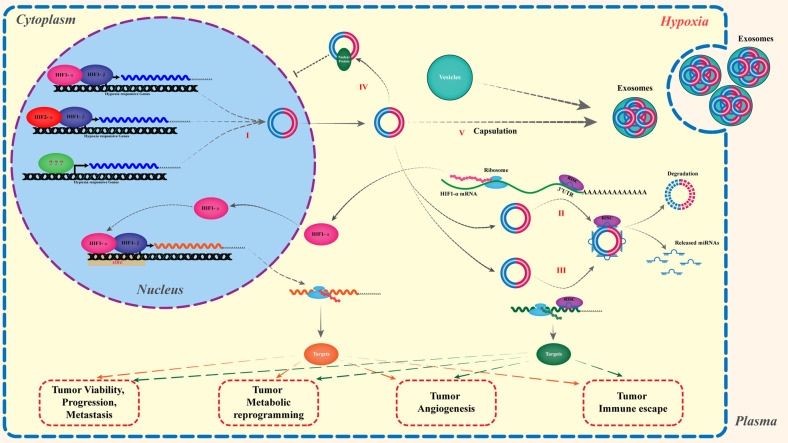
The roles and underlying mechanisms of hypoxia-responsive circRNAs played in tumor hypoxic environment. I: Biogenesis of cirRNAs under hypoxia. The hypoxia-responsive circRNAs may be induced in either HIFs-dependent or independent manners. II: Some hypoxia-responsive circRNAs can reversely regulate the expression of HIFs by serving as miRNA sponge. III: Some hypoxia-responsive circRNAs act as miRNAs sponge to regulate the expression of hypoxia-responsive genes (not HIFs). IV: A few hypoxia responsive circRNAs can decoy nuclear proteins by inhibiting their nucleus translocation. For instance, circZNF292 binds SOX9 protein in cytoplasm to inhibit its nuclear translocation. V: Some hypoxia-responsive circRNAs can be encapsulated in exosomes. For example, hsa-circ-0048117 is significantly enriched in and secreted by exosomes of hypoxia pre-challenged esophageal squamous cell carcinoma (ESCC) cells and contributes to M2 macrophage polarization. (See the text for details).

Most of recent works on hypoxia-responsive circRNAs of tumors mainly focused on the relationships between circRNAs and HIFs or the hypoxia responses per se, given that the HIFs complexes are the central mediator of tumor responses to hypoxia. In spite of that HIFs-dependent or independent regulated circRNAs exist, the transcription of circRNAs is regulated by HIFs, especially HIF1α, to aid the tumor responses to hypoxia. For example, of the hypoxia-induced circRNAs in human umbilical venous endothelial cells (circZNF292, circAFF1, circTHSD1, circDENND4C, circSRSF4 and circFOXJ3), only circDENND4C expression is HIF1α-dependent in breast cancer cell lines after hypoxia induction, which sponges miR-200b and miR-200c to promote breast cancer cell glycolysis, migration and invasion [26, 27]. Another hypoxia-responsive circRNA-circHIPK3 is a HIF-2α-dependent circRNA in a hypoxic resistant gastric cancercell lines (HRGC-R), which can directly sponges miR-338-3p and miR-653-5p to promote HRGC cells migration and invasion via increasing the expression of neuropilin 1 (NRP1) and activating the downstream ERK and AKT pathways [28]. In addition, has-circRNA-403658, formed via back-spliced between the 1st and 4th exon of its host gene-ZNF292, exhibits HIF1α-dependent expression in bladder cancer cells under hypoxia, and promotes bladder cancer cell growth by reducing cell apoptosis and activating LDHA-mediated aerobic glycolysis [29]. However, only a few HIFs-regulated circRNAs have been identified in hypoxic tumor, and these include circDENND2A in gliomas [30], circEPHB4 (hsa-circ-0001730) in hepatocellular carcinomas [31], hsa_circ_0008193 and circSETDB1 (hsa-circ-0003439) in lung adenocarcinoma [32, 33], et al. The detailed biogenesis of HIFs-dependent circRNAs under hypoxia remains unclear right now (Table 1, Fig. 3).

**Table 1:** The HIF-1α dependent or independent regulation of hypoxia-responsive circRNAs in hypoxic tumor microenvironment.

Hypoxia-responsive circRNAs	Status upon hypoxia	HIF involvement	Cancer types	Clinical association	Functional impact	Interactor	Target/effect	Mechanistic Classification	Refs
circDENND4C	Up-regulated	HIF-1α dependent	Breast cancer	Associated with tumor node metastasis stage lymph node metastasis and tumor size	Glycolysis↑ Migration↑ Invasion↑	miR-200b miR-200c	Increase glucose consumption, Lactate production, HK2 protein level, Migration, Invasion.	Sequestration of miRNAs (Down-regulation of miR-200b and miR-200c-mediated repression of glycolysis, migration and invasion)	[27]
circZNF292 (circRNA-403658)	Up-regulated	HIF-1α dependent	Bladder cancer	Promoting cancer progression	Cell growth↑ Glycolysis↑ Angiogenesis↑ Apoptosis↓	LDHA	LDHA promotor	Transcriptional regulation promoting the activity of the LDHA promoter	[29]
circDENND4C	Up-regulated	HIF-1α dependent	Breast cancer	Positively correlated to tumor size	Proliferation↑	N.D.	N.D.	Unclear mechanism	[46]
circHIPK3	Up-regulated	HIF-2α dependent	Gastric Cancer	Shorter overall survival (OS)	Migration↑ Invasion↑	miR-653-5p miR-338-3p	Activation of downstream ERK and AKT pathways	Sequestration of miRNAs (Down-regulation of miR-653-5p and miR-338-3p-mediated repression of NRP1 expression)	[28]
circSETDB1 (hsa-circ-0003439)	Up-regulated	HIF-1α pathway related	Lung adenocarcinoma (LUAD)	Correlated with T stage and lymph node metastasis	Migration↑ Invasion↑ Proliferation↑	miR-7	Promoted lung cancer EMT	Sequestration of miRNAs (Down-regulation of miR-7-mediated repression of Sp1 expression)	[33]
circDENND2A	Up-regulated	HIF-1α related	Glioma	Enhances glioma metastases	Migration↑ Invasion↑	miR-625-5p	HIF1α or SOX2	Sequestration of miRNAs (Down-regulation of miR-625-5p-mediated repression of HIF1α or SOX2 expression)	[30]
circ_0008193	Down-regulated	HIF-1α related	Lung adenocarcinoma (LUAD)	Correlated with tumor size and lymph node metastasis	Cell viability↓ Glucose uptake↓ Lactate production↓ Migration↓ Invasion↓	miR-1180-3p	Inhibited LUAD cell proliferation, migration, invasion, and Warburg effect under hypoxia in vitro and in vivo	Sequestration of miRNAs (Down-regulation of miR-1180-3p-mediated repression of TRIM62 expression)	[32]
circ-EPHB4 (hsa-circ-0001730)	Down-regulated	HIF-1α related	Hepatocellular carcinoma	Negatively correlated with tumor weight, size, and metastasis foci	tumorigenesis↓ Tumor development↓ Metastasis↓	N.D.	HIF1α	Unclear mechanism	[31]
circELP3	Up-regulated	HIF-1α independent neither the HIF-associated pathway nor increased circulation	Bladder cancer	Associated tumor grade and lymph node metastasis	Proliferation↑ Self-renewal capacity↑ Cisplatin resistance↑	OCT4,SOX2 Nanog	Promote cisplatin resistance	Unclear mechanism	[34]
circZNF292	Up-regulated	HIF-1α independent	Hepatoma cells	N.D.	Proliferation↑ Vasculogenic mimicry↑ Radioresistance↑	SOX9	Enhanced Wnt/β-catenin pathway activity	Sequestration of Protein Binding to SOX9 protein in cytoplasm and inhibited its nuclear translocation	[47]
circHIF1A	Up-regulated	HIF-1α independent	Cancer-associated fibroblasts (CAFs) in breast cancer	N.D.	Cell proliferation↑ Stemness↑	miR-580-5p	Promoted breast cancer cell proliferation and stemness	Sequestration of miRNAs (Down-regulation of miR-580-5p-mediated repression of CD44 expression)	[36]

PS: *N*.*D*. Not Determined.

### HIFs-independent regulation of hypoxia-responsive circRNAs in tumors

Compared to HIFs-dependent circRNAs, few HIFs-independent circRNAs have been identified under hypoxia. For instance, in one study, circELP3 was obviously upregulated in bladder cancer cells lines (T24 and 5637) under hypoxia, while the level of host genes ELP3 expression did not change. Even when HIF1α and HIF-2α were silenced, the circELP3 was also induced by hypoxia. In addition, the elevated level of circELP3 did not fall when circular RNA circulation assistant factors (DHX9, ADAR1 and QKI) were silenced [34]. By contrast, has-circRNA-403658 (cZNF292) is a HIF1α-induced circRNA in bladder cancer [29], however, it serves as a HIF1α-independent circRNA in hypoxic hepatoma cells [35]. In addition, circHIF1A (circ_0032138) was upregulated in the exosomes, derived from hypoxic Carcinoma-Associated Fibroblasts (CAFs) with time-dependent manner, however it was not influenced by HIF-1α inhibition [36] (Table 1, Fig. 3).

### Hypoxia-responsive circRNAs reversely regulate HIFs to facilitate tumor cells to adapt to hypoxia stress

Under hypoxia, HIFs can accumulate on the HRE of the promotor of their downstream genes to swiftly trigger an adaptive genomic landscape to facilitate cancer progression. Several circRNAs have been identified to regulate the expression of HIFs, especially for HIF-1α, thus contributing to cancer progression, invasion, metabolism reprogramming, immune escape, et al. For instance, circRNA_100859 absorbs miR-217 to release the inhibition of HIF-1α expression, facilitating colon cancer progression [37]. Moreover, circRNF20 (hsa_circ_0087784), circulated from RNF20 gene exon-3 to exon-5, has been newly identified in breast cancer samples, and promotes the progress and glycolysis of breast cancer by abrogating the miR-487a-mediatied inhibition of the HIF-1α/HK2 axis [38]. In addition, hypoxia-induced circ_0000977 facilitates the immune escape of pancreatic cancer cells by absorbing miR-153 to abrogate the inhibition of HIF-1α and ADAM10 [39]. Except of these circRNAs mentioned above, a number of other circRNAs has been identified to modulate the expression of HIFs, especial for HIF-1α, in favor of the progression and glycolysis of tumor cells under normoxia or hypoxia, which include circNRIP1 [40], circHIPK3 [41], CircZFR [42], circ_03955 [43], hsa-circ-0000211 [44], circPIP5K1A/hsa_circ_0014130 [45], and so on (Table 2, Fig. 3).

**Table 2:** The circ RNAs involving into the regulation of HIF-1α in hypoxic tumor microenvironment.

Hypoxia-responsive circRNAs	Status upon hypoxia	HIF involvement	Cancer Types	Clinical association	Functional Impact	Interactor	Target/Effect	Mechanistic classification	Refs
circ0000977	Up-regulated	N.D.	Pancreatic cancer	N.D.	Immune escape↑	miR-153	Faciliating HIF1A-mediated immune escape of PC cells	Sequestration of miRNAs (Down-regulation of miR-153-mediated repression of HIF-1α and ADAM10 expression)	[39]
circNRIP1	Up-regulated	N.D.	Gastric cancer	N.D.	5-fluorouracil (5-FU) resistance↑ Glucose consumption↑ Lactate production↑ Glucose-6-phosphate (G6P)↑	miR-138-5p	The expression of HIF-1α and modulation of HIF-1α-dependent glycolysis	Sequestration of miRNAs (Down-regulation of miR-138-5p-mediated repression of HIF-1α expression)	[40]
circHIPK3	Up-regulated	N.D.	Cervical cancer	N.D.	Cell growth↑ Metastasis↑	miR-338-3p	The expression of HIF-1α and regulating HIF-1α mediated EMT	Sequestration of miRNAs (Down-regulation of miR-338-3p-mediated repression of HIF-1α expression)	[28]
circZFR	N.D.	N.D.	Breast cancer	Predicted poor prognosis	Cell viability↑ Colony formation↑ Migration↑ Invasion↑ Glycolysis↑	miR-578	Regulate of the miR-578/HIF1A axis to promote BC malignant progression	Sequestration of miRNAs (Down-regulation of miR-578-mediated repression of HIF-1α expression)	[42]
circRNA_100859	N.D.	N.D.	Colon cancer	Associated with Tumor-Node-Metastasis (TNM) stage, histological grade, and KRAS mutations, and also showed high diagnostic and prognostic value	Cell proliferation↑ Apoptosis↓	miR-217	Contributing HIF-1α-dependent colon cancer progression	Sequestration of miRNAs (Down-regulation of miR-217-mediated repression of HIF-1α expression)	[37]
circRNF20 (hsa_circ_0087784)	N.D.	N.D.	Breast cancer	Predicted the poor clinical outcome; correlated with lymph node metastasis and tumor size	Proliferation↑ Warburg effect↑	miR-487a	Facilitates HIF-1α-dependent the transcription of HK2	Sequestration of miRNAs (Down-regulation of miR-487a-mediated repression of HIF-1α expression)	[38]
circ_03955	N.D.	N.D.	Pancreatic cancer	Poor clinical outcomes	Proliferation↑ Apoptosis↓ Glycolysis↑	miR-3662	Facilitates HIF-1α-dependent tumorigenesis and Warburg effect	Sequestration of miRNAs (Down-regulation of miR-3662-mediated repression of HIF-1α expression)	[43]
hsa-circ-0000211	N.D.	N.D.	Lung adenocarcinoma (LUAD)	Positively correlated with the distant metastasis	Migration↑ Invasion↑	miR-622	Facilitates HIF-1α-dependent migration and invasion	Sequestration of miRNAs (Down-regulation of miR-622-mediated repression of HIF-1α expression)	[44]
circPIP5K1A (hsa_circ_0014130)	N.D.	N.D.	Non‐small cell lung cancer (NSCLC)	N.D.	Metastasis↑ Proliferation↑	miR‐600	The expression of HIF-1α and regulating HIF-1α mediated EMT	Sequestration of miRNAs (Down-regulation of miR-600-mediated repression of HIF-1α)	[45]

PS: *N*.*D*. Not Determined.

## The impact of hypoxia-responsive circrnas on cancer responses to hypoxia

Under oxygen deprivation, hypoxia-responsive circRNAs are either up- or downregulated to allow tumor cells adapt to such microenvironmental stress, with which tumor cells become more aggressive, enhanced glycolysis, invasive angiogenesis and immune escape in HIFs-dependent or independent manners.

### Hypoxia-responsive circRNAs regulate hypoxic tumor progression and metastasis

Hypoxia per se, or HIFs is sufficient to induce the Epithelial-Mesenchymal Transition (EMT) and invasion of multiple cell types by direct or indirect ways. Usually, HIFs can promote EMT by acting directly on several EMT transcription factors such as ZEB1, Snail, and Twist, or indirectly via a number of signaling pathways, including Notch, TGF-β, Wnt, Hedgehog, etc. [46, 47]. It has been demonstrated that several hypoxia-responsive circRNAs play important roles in hypoxic tumor aggression and metastasis. For instance, hypoxia-responsive circ-Erbin facilitates the aggression and metastasis of colorectal cancer (CRC) by accelerating the cap-independent protein translation of HIF-1α [48]. Moreover, the hypoxia-responsive circCDR1as contributes to oral squamous cell carcinoma (OSCC) survival by activating AKT and ERK½ pathways and suppressing rapamycin (mTOR) activity, which enhances autophagy, cells’ viability, but inhibits apoptosis [49]. Furthermore, circ-133, derived from hypoxic colorectal cancer exosomes, promotes the metastasis of CRC via the miR-133a/GEF-H1/RhoA axis [50].

### Hypoxia-responsive circRNAs regulate the metabolic reprogramming of hypoxic tumors

Usually upon hypoxia, the primary cellular metabolic strategy can swiftly shift from predominantly mitochondrial respiration towards glycolysis to maintain ATP levels, which can be regulated by HIFs-dependent or independent expression of glycolytic enzymes [51–53]. Several circRNAs have been identified to modulate such reprogramming to promote cancer cell growth and invasion under hypoxic stress. For example, has-circRNA-403658, a hypoxia-responsive circRNA in bladder cancer, promotes LDHA-mediated aerobic glycolysis and growth in bladder cancer cell [29]. Moreover, circMAT2B, highly increased in HCC tissues, promotes glycolysis under hypoxia via the circMAT2B/miR-338-3p/PKM2 axis, which is correlated with poor prognosis [54]. Furthermore, the novel circRNF20 (hsa_circ_0087784) is expressed at high levels in breast cancer (BC), and promotes the proliferation and glycolysis of BC cells via the miR-487a/HIF-1α/HK2 axis [38]. In addition, circDENND4C and hsa_circ_0001982 in breast cancer [27, 55], circ_0000376 in Non-Small Cell Lung Cancer (NSCLC) [56], circSLAMF6 in gastric cancer [57], and circ_0008450 in hepatocellular cancer [58] have been demonstrated to be induced by hypoxia, and play important roles in the glycolysis and progression of hypoxic cancer.

### Hypoxia-responsive circRNAs mediate hypoxic tumor angiogenesis and immune escape

During angiogenesis, new blood vessels sprout from preexisting vessels, thus remodeling and expanding the primary vascular networks, which greatly aids progression of hypoxic tumors. The formation of vascular-like structures in endothelial cells is the very important step to angiogenesis of hypoxia tumor [1, 59]. Recently, several hypoxia-responsive circRNAs have been identified to play essential roles in hypoxic angiogenic signaling. For instance, silencing of cZNF292 suppresses tube formation in glioma U87MG and U251cells by downregulating the levels of VEGFR-1/2, phosphorylated VEGFR-1/2 (p-VEGFR-1/2), VEGF-A, EGF, TGF-β1 and EGFR [60]. Such silencing inhibits the vasculogenic mimicry (VM) of hepatoma cells by increasing SOX9 nuclear translocation both in vitro and in vivo [35]. Additionally, circ-Erbin accelerates the cap-independent protein translation of HIF-1α, increasing the number of microvessels of CRC in vivo [48]. Tumor immune escape refers to the phenomenon of tumor cells growing and metastasizing via various mechanisms to avoid recognition and attack by the immune system [1]. Hypoxia-induced circ_0000977 has been identified to modulate immune escape of pancreatic cancer cells via the miR-153/HIF-1α axis [39]. Moreover, hsa-circ-0048117, enriched in exosomes from hypoxia pre-challenged esophageal squamous cell carcinoma (ESCC) cells, facilitates the M2 macrophage polarization and promotes the proliferation and metastasis of ESCC cells by absorbing miR-140 [61].

### Relevance of hypoxia-responsive circRNAs to several clinical features of cancer

The unique expression patterns (universality and tissue/cell specificity) and special characteristics (conservation, and stability) of circRNAs render them ideal potential biomarker candidates, which may greatly aid cancer diagnosis, monitoring of progression and determination of prognosis [1]. Due to the fact that larger tumors suffer more hypoxic damage than smaller ones and normal tissues, hypoxia-responsive circRNAs can serve as new diagnostic biomarkers that reflect tumor volume, TNM stage, lymphatic infiltration and distant metastasis. For instance, significantly increased in the serum of patients with HCC patients, circ_0008450 has relevance with tumor volume, TNM stage, lymphatic and distant metastasis of these patients; So it can be used to distinguish such patients from the cohort in ROC analysis(AUC = 0.97) [58]. Moreover, the level of circDENND4C level is associated with tumor volume, TNM stage, lymphatic infiltration and distant metastasis of breast cancer (BC), in spite of differential expression in the four subtypes (TNBC, HER2, Luminal A and B) [27]. Furthermore, higher circELP3 level in bladder cancer patients is positively correlated with advanced tumor type, with either tumor stage higher than T2 or lymph node metastasis [34].

Secondly, hypoxia-responsive circRNAs have been used as prognostic biomarkers to predict the overall survival of tumor patients and their sensitivities to radio-chemotherapy. For example, higher circ_0000376 expression is not only closely associated with the tumor volume, TNM stage, and lymphatic metastasis of non-small lung cancer, but also correlated with the lower overall survival rate of such patients [56]. Moreover, higher circRNF20 expression is closely associated with tumor volume and lymphatic metastasis, and indicated the poor overall survival of breast cancer (BC) patients [38]. Furthermore, hypoxia-induced circNRIP1 enhances the resistance of gastric cancer (GC) to 5-FU by modulating HIF-1α-dependent glycolysis [40]. Additionally, silencing of cZNF292 suppresses hypoxic hepatoma radioresistance by reducing Wnt/β-catenin pathway activity both in vitro and in vivo [35].

In addition, as exosomes have been viewed as critical mediators of intercellular communication, circRNAs in hypoxic exosomes have been associated with various biological and clinical properties of tumors recently. For instance, hypoxia-induced circSETDB1, enriched in serum exosomes of lung adenocarcinoma (LUAD), was closely associated with T stage and lymph node metastasis of such patients [33]. What’s more, circHIF1A, in the hypoxic exosomes of cancer-associated fibroblasts (CAFs), has been associated with breast cancer’s stem cell properties [36]. Furthermore, enriched in the exosomes of CRC patients’ plasma, hypoxia-responsive circ-133 has been found to be correlated with disease progression of these patients [50]. Additionally, derived from hypoxic pre-challenged exosomes, hsa-circ-0048117 plays key roles in remodeling the microenvironment of esophageal squamous cell carcinoma (ESCC) and promoting cancers’ proliferation and metastasis [61].

## Summary and perspectives

Circular RNAs are attracting increasing attention of researchers, based on their numerous properties and various biological functions. Recently, as novel participants in hypoxia-induced non-coding RNA transcriptomics, more and more studies reveal that hypoxia-responsive circRNAs play important roles in regulation of cancer hypoxic responses, which make it serve as molecular biomarkers for disease diagnosis, prognosis and evaluation of therapeutic effect. However, such researches remain in its infancy.

More hypoxia-responsive circRNAs’ targets and regulatory modules are yet to be discovered. For instance, many scholars currently focus on the reciprocal inhibitions between circRNAs and miRNA, but the assembly between circRNAs and proteins, the roles of their secondary and more advanced structures and their roles in epigenetic modification of genome under hypoxia remain unknown. Moreover, except HIFs-dependent responses, it is unknown how circRNAs affect the unfolded protein response (UPR) and mammalian target of rapamycin (mTOR) pathway, which act in parallel with, or even substitute for HIFs activity under hypoxia. Therefore, further works about modulation of hypoxia-responsive circRNAs on above two signaling pathways under hypoxia are needed to be explored in the future. Furthermore, although there have been great advances in identification and role of hypoxia-responsive circRNAs under hypoxia, many questions concerning biogenesis of circRNAs and their roles of post-transcriptional regulation remain to be explored. For instance, how are circRNAs generated from their linear RNA counterparts under hypoxia? Can hypoxia-responsive circRNAs be translated in peptides or proteins, and if so, what are their functions? How are such translation products ultimately degraded under hypoxia? and so forth.

In summary, we have reviewed the regulatory roles of hypoxia-responsive circRNAs and how they mediate hypoxic responses, as well as their relevance with clinical features, which serve as valuable clinical biomarkers for facilitating disease diagnosis, evaluating prognosis and estimating therapeutic effects. However, the link between hypoxia-responsive circRNAs and hypoxia per se must be further explored. In the future, we are convinced that more and more hypoxia-responsive circRNAs will be discovered and their regulatory roles in hypoxia will be understood in increasing detail.

## Supplementary information

Author Contribution Statement

Authors information

CDDIS-22-1633R-clean manuscript

CDDIS-22-1633R-checklist

CDDIS-22-1633R_Revision_certificate

## Data Availability

All data generated or analyzed during this study are included in this published article and supplementary materials.
