# Supplementary material for: Hypoxia-responsive circRNAs: A novel but important participant in non-coding RNAs ushered toward tumor hypoxia
Source: Cell Death Dis. 2022 Aug 1;13(8):666. doi: 10.1038/s41419-022-05114-y (PMC9343381; doi:10.1038/s41419-022-05114-y)
Supplement: Supplementary file 1 — Author Contribution Statement [file 41419_2022_5114_MOESM1_ESM.docx]

**Author Contribution Statement**

**Benzheng Jiao and Shanshan Liu** contribute equally to collect the related papers and finish the manuscript.

**Hongguang Zhao and Yuying Zhuang** drafted the tables and made the figures for the manuscript.

**Shumei Ma and Chenghe Lin** made critical revisions for this review.

**Xiaodong Liu and Jifan Hu** participated in the design of this review and gave constructive guidance.

All authors read and approved the final manuscript.
