# Supplementary material for: Hypoxia-responsive circRNAs: A novel but important participant in non-coding RNAs ushered toward tumor hypoxia
Source: Cell Death Dis. 2022 Aug 1;13(8):666. doi: 10.1038/s41419-022-05114-y (PMC9343381; doi:10.1038/s41419-022-05114-y)
Supplement: Supplementary file 2 — Authors information [file 41419_2022_5114_MOESM2_ESM.docx]

**Full names and Email address**

Benzheng Jiao, jiaobenzheng@jlu.edu.cn

Shanshan Liu, liuss8888@jlu.edu.cn

Hongguang Zhao, zhaohg@jlu.edu.cn

Yuying Zhuang, zhuangyy1997@163.com

Shumei Ma, [shmm2001@126.com](mailto:shmm2001@126.com)

Chenghe Lin, linch@jlu.edu.cn

Jifan Hu, jifan@stanford.edu

Xiaodong Liu, liuxd2014@ 126.com
