# Supplementary material for: Hypoxia-responsive circRNAs: A novel but important participant in non-coding RNAs ushered toward tumor hypoxia
Source: Cell Death Dis. 2022 Aug 1;13(8):666. doi: 10.1038/s41419-022-05114-y (PMC9343381; doi:10.1038/s41419-022-05114-y)
Supplement: Supplementary file 5 — CDDIS-22-1633R_Revision_certificate [file 41419_2022_5114_MOESM5_ESM.pdf]

Textcheck Certificate

Refnum:	22062103
Title:	Hypoxic responsive circRNAs
Date:	2022/06/25

We hereby certify that Textcheck has checked and corrected the English in the manuscript named above.

A specialist editor with suitable professional knowledge (M.Sc. or Ph.D./M.D.) reviewed and corrected the English. An English language specialist subsequently checked the paper again. The first language of both editors is English.

Please direct any questions regarding this certificate or the English in the certified paper to: certified@textcheck.com (Please quote our reference number: '22062103')
